# Anatomical variations of the renal artery: a computerized tomographic angiogram study in living kidney donors at a Nigerian Kidney Transplant Center

**DOI:** 10.4314/ahs.v21i3.24

**Published:** 2021-09

**Authors:** Abayomi Aremu, Martin Igbokwe, Olalekan Olatise, Ahmad Lawal, Kester Maduadi

**Affiliations:** 1 Urology Division, Zenith Medical and Kidney Centre, Gudu, Abuja, Nigeria; 2 Nephrology Unit, Zenith Medical and Kidney Centre, Gudu, Abuja, Nigeria; 3 Urology Division, Ahmadu Bello University Teaching Hospital, Zaria, Kaduna State, Nigeria; 4 Radiology Department, Zenith Medical and Kidney Centre, Gudu, Abuja, Nigeria

**Keywords:** Renal artery, variations, living kidney donors

## Abstract

**Background:**

Understanding of the renal vascular anatomy is key to a safe and successful donor nephrectomy, which ultimately impacts on the renal graft function and survival in kidney transplant recipients.

**Objective:**

To report the various anatomical configurations of the renal artery identified in living kidney donors in a Nigerian kidney transplant institution.

**Materials and Methods:**

The computerized tomography angiograms of 100 consecutive living kidney donors were prospectively reviewed over an 18-month period. Anatomical variations of the renal arteries including accessory arteries and early divisions were noted. Duration of surgery and ischemic time were recorded intra-operatively. Data analysis was carried out using IBM SPSS version 20.

**Results:**

There were variations in renal artery configuration in 50 (50%) cases, 32% were accessory renal arteries while 18% were early branches of the renal artery. The classical bilateral solitary renal arteries were found in 50 (50%) of potential donors. There was statistically significant longer operating and ischemic time in donors with multiple renal arteries as compared with solitary arteries (p<0.05).

**Conclusion:**

There are a wide variety of renal artery configurations seen in potential kidney donors. The classical solitary renal artery remains the commonest and most favourable configuration for donor nephrectomy and transplantation.

## Introduction

The anatomy of the renal artery plays a key role in selection of kidney donors for a renal transplant program^1^. The ideal renal artery for ease of vascular anastomosis is one which is solitary, of good diameter and length. Each kidney is classically supplied by a single renal artery, which arises as a lateral branch of abdominal aorta, between the levels of 1^st^ and 2^nd^ lumbar vertebrae^2^, ^3^. The left renal artery is shorter in length while the longer right renal artery passes posterior to the inferior vena cava (IVC) to gain access to the kidney at the renal hilum. Renal arteries give branches to the adrenal glands, renal pelvis and proximal ureters. After entering the hilum, each artery divides into five segmental end arteries which do not freely anastomose with each other. This therefore means that injury to a segmental renal artery would cause a segmental renal infarction. Computerized Tomography Angiography (CTA) is the investigation of choice for identifying the renal arterial anatomy^4^. Studies have shown that the classical description of the renal vasculature, formed by one renal artery and vein are found in less than 25% of the population^5^, ^6^. There have also been controversies in literature with regards the number of renal arteries and their branches^7^–^9^. Most often encountered morphological variations of renal artery are its variable number and unusual branches originating from it^10^–^12^. These variations are sometime incidentally found during autopsy or surgical operation. Terminologies like supranumerary, supplementary and accessory have been used to describe the variable configurations of the renal artery. According to Sampaio and Passos, these arteries are referred to as multiple, since they are segmental vessels for the kidneys, without anastomoses between themselves and they should be named according to the territory supplied by them as- hilar, superior polar and inferior polar^13^. The knowledge of the possible variations in the configuration of the renal arteries is necessary for surgical management during donor nephrectomy, repair of abdominal aorta aneurysm, other retroperitoneal urological procedures and angiographic interventions.^14^–^16^

The aim of this study is to report the various anatomical configurations of the renal arteries in a cohort of randomised healthy kidney donors using computerized tomography angiogram.

## Materials and Method

This was a prospective, cross-sectional, hospital-based study conducted on 100 healthy living kidney donors in Zenith Medical and Kidney Center (ZMKC), Gudu, Abuja over an 18- month period (January 2018 to June 2019). Patients who were being planned for donor nephrectomy haven been found compatible with a recipient were recruited for this study. During the study period, the CT angiographic images were reviewed independently by two consultant urologists and a radiologist. All CT angiographic images were performed in the radiology unit of ZMKC using the unit protocol. The images were studied for the number of renal arteries originating from the abdominal aorta, the presence of early divisions into segmental arteries and presence of accessory arteries. For the purpose of this study, any division of the renal artery within 1cm from its origin on the abdominal aorta was considered an early division while any other artery arising from the aorta or its branches to supply the kidney other than the main renal artery was termed an accessory artery. Tiny cortical branches were not taken into account as accessory renal arteries. Donor nephrectomies were all performed using a flank, extra-peritoneal approach using 11^th^ rib-transcostal or subcostal incisions while the recipient kidney transplant is performed in supine position via and extra-peritoneal approach using a Gibson incision. The findings were analyzed in both kidneys per patient and data was entered into a pre-designed proforma. Descriptive statistics were used to express the data with continuous variables summarized using arithmetic mean and standard deviation, while the categorical variables were summarized as proportions and frequencies. A p-value of less than 0.05 was considered significant. Data analysis was carried out using IBM Statistical Package for Social Sciences (SPSS) version 20.

## Result

A total of 100 patients were successfully recruited for this study. Their ages ranged from 18 to 53 years with a mean age of 31.2±7.7years. Males accounted for 88% while the rest were females (Table 1). Ninety-eight (98%) of the donors eventually underwent donor nephrectomy while 2 of the potential donors could not proceed with surgery due to sudden death (1) and a cerebrovascular accident (1) in the recipients few hours-days to surgery.

**Table 1:** Socio-demographic distribution of living kidney donors

Variable	n
(%)	
**Sex**	
Male	88 (88)
Female	12 (12)
**Marital status**	
Single	79 (79)
Married	21 (21)
**Age Range**	
11–20	4 (4)
21–30	58 (58)
31–40	30 (30)
41–50	6 (6)
51–60	2 (2)
61–70	1 (1)
**Total**	**100 (100)**

**Figure 1:**
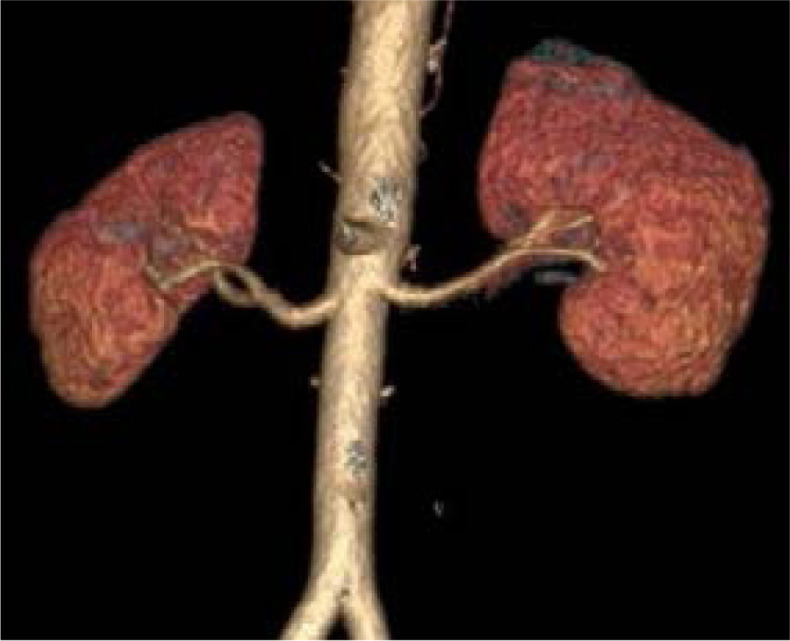
Showing single renal artery on both sides

**Figure 2:**
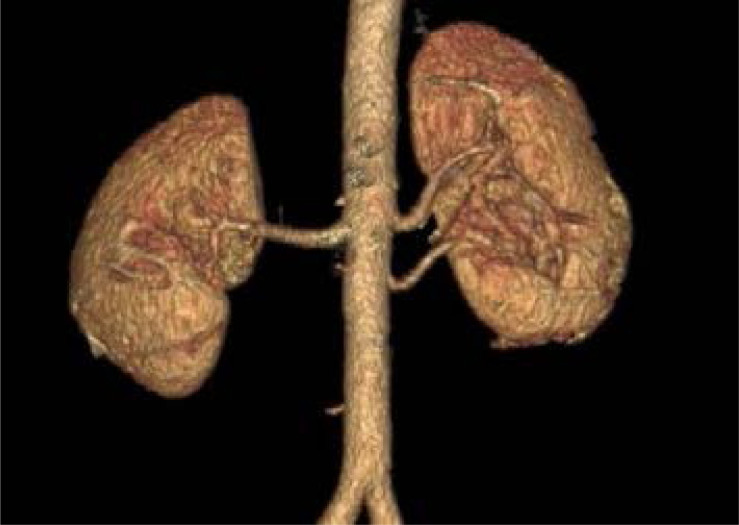
Showing an accessory hilar arteryto the left kidney

Of the 100 CTAs studied, we observed that 50 (50%) had the classical bilateral vascular renal arterial anatomy of a solitary renal artery with no accessory branch or early division (Figure 1). Among the other 50 patients, 32 (32%%) had either unilateral or bilateral accessory renal arteries while 18 (18%) had unilateral or bilateral early divisions of the renal artery. An accessory renal artery was found on the right side in 10 patients (20%) and left side in 12 cases(24%) respectively (Figure 2).

**Figure 3:**
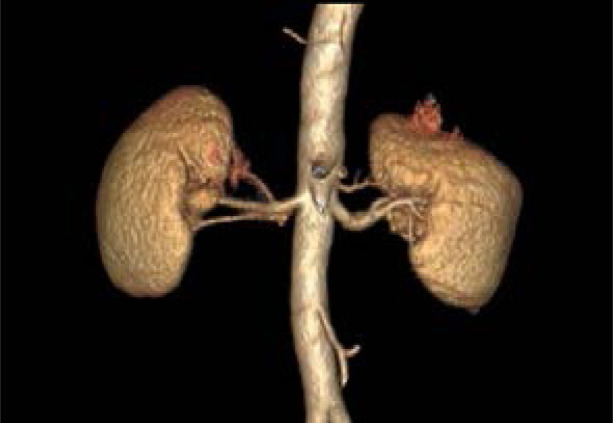
Bilateral accessory renal arteries

**Figure 4:**
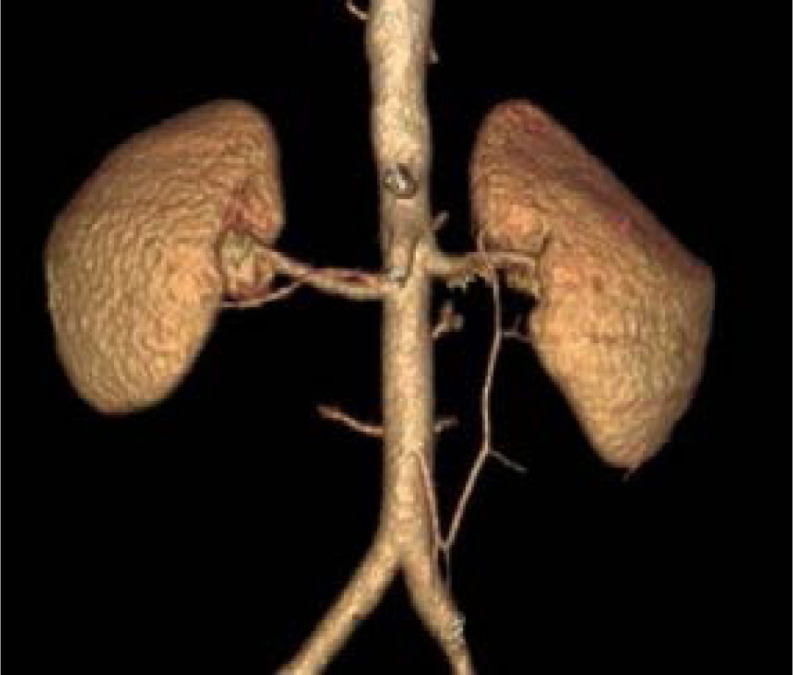
Unusual bilateral accessory renal arteries

Bilateral accessory vessels were found in another 10 patients(Figure 3) with various rare variants seen in our series (Figure 4). Out of the 18(18%) cases of early branching artery, 9% and 8% were found on the right and left side respectively while only one case of bilateral early branching was seen (Table 2).

**Table 2:** Distribution of Renal Artery configuration findings on CT Angiogram

VARIABLES	N (%)
**Normal and variations of renal artery Normal and variations of** renal artery (N = 100)	

Normal (Solitary renal arteries bilaterally) Variations	50 (50) 50(50)

**Distribution of variations in renal artery (N = 100)**	

Accessory renal artery Early branching of renal artery	32 (32) 18 (18)

**Distribution of accessory renal arteries**	

Right accessory arteries Left accessory artery	10 (10) 12(12)

**Bilateral accessory renal arteries**	

Single accessory arteries bilaterally Double accessory renal arteries bilaterally	9 (9) 1 (1)
**Anatomical distribution of accessory renal arteries (N = 100)**	
Single hilar	
Right Side	80 (80)
Left Side	76 (76)
Double hilar	
Right	6 (6)
Left	11 (11)
1 hilar and 1 Superior polar artery	
Right	5(5)
Left	2 (2)
1 hilar and 1 Inferior polar artery	
Right	9 (9)
Left	10 (10)
1 Hilar, 1 Superior polar and 1 Inferior polar artery	
Right	-
Left	1 (1)

The presence of both accessory renal arteries and early divisions were higher among kidneys of male donors when compared to female donors (p= 0.002 and p=0.003 respectively).

**Figure 5:**
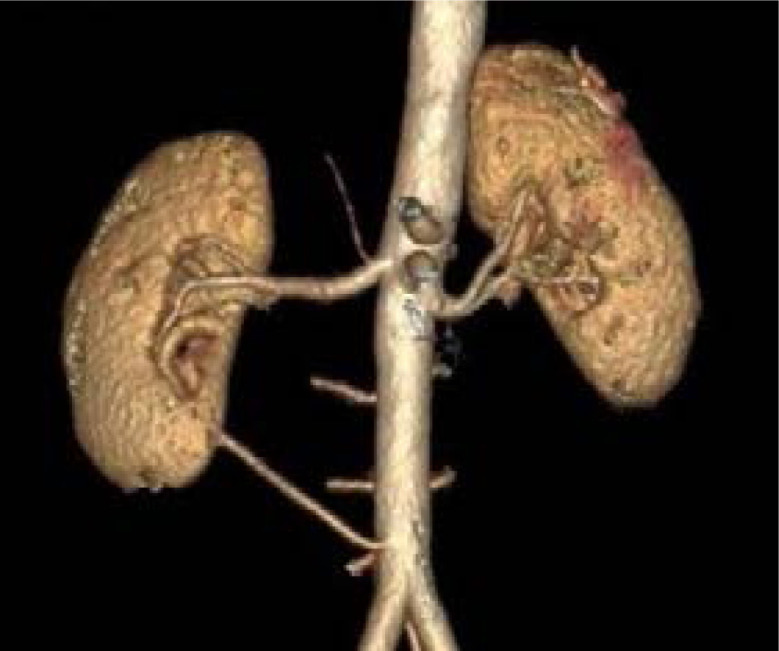
Co-existence of accessory right renal artery and left early division of renal artery

There was a co-existence of early division and accessory renal arteries in 2 patients (Figure 5).

In the 10 cases where there were bilateral accessory renal arteries, the side with larger diameter accessory renal artery was selected for donor nephrectomy. These renal allografts when extracted intra-operatively required meticulous bench vascular surgery. 6 of allografts had bench artero-arterostomy in end-to-side fashion to produce a common stump using prolene 6/0 sutures while 4 others had 2 separate annastomosis of the renal arteries to the external iliac artery in end-to-side fashion. All kidneys had good perfusion and renal function on the table. There was significantly longer surgery time, warm and cold ischemic time in renal allografts with multiple arteries (p<0.05) (Table 3). These findings significantly affected our decision of the renal unit to harvest from the donor for transplantation. There was an inadvertent transection of an early division of the renal artery in 1 donor nephrectomy allograft with consequent devascuarisation of about 20% of the allograft requiring bench re-anastomosis of the transected segments of the artery with satisfactory outcome.. There was a significantly longer total surgery time, warm and cold ischemic time among kidney donors with solitary renal arteries when compared to multiple (accessory/ early branches) renal arteries (Table 3).

**Table 3:** Breakdown of Mean durations of surgery time and ischemic time

Variable	Mean duration (minutes) +/- Standard deviation	P value
**Total Surgery time**		
Solitary renal artery	115 +/- 15.4	**0.010**
Multiple renal arteries	135+/- 23.0	

**1^st^ Warm Ischemic time**		
Solitary renal artery	2.5+/- 0.6	**0.014**
Multiple renal arteries	4.5+/- 1.2	

**Cold ischemic time**		
Solitary renal artery	22+/- 16.5	**0.005**
Multiple renal arteries	45+/-20.0

**2^nd^ Warm Ischemic time**		
Solitary renal artery	43+/- 28.5	**0.007**
Multiple renal arteries	55+/-20.0

## Discussion

The knowledge of the detailed anatomy of the renal arteries plays an important role in planning major surgical procedures like live donor nephrectomy. The need for CT angiographic studies can therefore not be over-emphasized in this regards. Presence of an accessory renal artery connotes a more challenging surgical prospect for both the donor nephrectomy and allograft implantation surgeons. In cases where the kidney with an accessory renal artery cannot be avoided for donor nephrectomy (especially when they occur bilaterally), there is a need for meticulous dissection of the accessory arteries to ensure good length for ease of anatomosis. This will also translate to a longer cold and second warm ischemic time as there may be need for a double-barrel bench anastomosis of the accessory arteries or performing multiple vascular anatomosis with the iliac artery of choice on the recipient. Considering these challenges, it is usually the surgeons's preference to harvest a renal unit with the classical configuration of the renal artery. Our study revealed that 1 in 2 patients had the classical single bilateral renal arteries which was similar to findings by Kumaresan et al where 49 % had the normal anatomy^17^. The high incidence of renal artery variations regarding their origin and number as found in the index study has been similarly reported by many researchers 7–9. The various types of accessory renal arteries, their positions, method of entry to the kidney and its segmentation were studied extensively by Sykes (1963)^18^. Obstruction to any of these vessels leads to infarction of the segment of the kidney supplied.^13^ Due to the important clinical correlation of these real arterial variations, terminologies like hilar, superior polar and inferior polar renal arteries have been described in literature in order to better qualify them.

Similar to our finding on the prevalence of early branching and accessory renal arteries (18% & 32% respectively), Budhiraja et al in an Indian cadaveric study and Kumaresan et al reported close results. This however slightly differed from a study by Gumus et al who reported early division in 27% and accessory renal artery in 27%^19^. This may however be as a result of racial differences asrenal artery variations are more common in Africans than Indians^20^, ^21^. Bilateral early branching which was seen in only 1% in the index study is also a rare finding in literature (ref). Intra-operatively during donor nephrectomy and renal pelvis surgeries, superior and inferior polar extra hilar branches can be injured during mobilization^13^. Inferior polar artery injuries can be a cause of ureteropelvic junction obstruction10. Nowadays, allografts with multiple renal arteries have become a necessity to maintain donor pool^22^, but its outcome is still a matter of discussion. Some surgical scholars opine that multiple renal arteries increase the chances of acute rejection and poor graft functions 23, 24, while others like Benedetti et al^25^ did not find significant difference with regard to acute rejection rate in grafts with single versus multiple arteries. It is however agreed generally that allografts with multiple renal arteries have a higher risk of renal artery stenosis 26 and technical difficulties for surgeon performing transplant operation^7^, ^27^

## Conclusion

Fifty percent of living kidney donors have either early divisions or accessory renal arteries .It has become increasingly important for kidney transplant surgeons to familiarize themselves with the anatomical variations of the renal artery and its correlation with surgery.

## Recommendation

There maybe a need to carry out a larger multi-centered and international study in order to assess a larger population of donors so as to have a more robust and representative result.
